# Imposter or knight in shining armor? Pelvic circumferential compression devices (PCCD) for severe pelvic injuries in patients with multiple trauma: a trauma-registry analysis

**DOI:** 10.1186/s13049-023-01172-z

**Published:** 2024-01-16

**Authors:** H. Trentzsch, R. Lefering, U. Schweigkofler, the TraumaRegister DGU

**Affiliations:** 1 Institut für Notfallmedizin und Medizinmanagement (INM), Klinikum der Universität München, Ludwig-Maximilians-Universität, Schillerstr. 53, 80336 Munich, Germany; 2 Institute for Research in Operative Medicine (IFOM), Faculty of Health, University Witten/Herdecke, Cologne, Germany; 3 Department of Trauma and Orthopedic Surgery, BG Unfallklinik Frankfurt Am Main Friedberger, Landstr. 430, 60389 Frankfurt am Main, Germany; 4 Committee on Emergency Medicine, Intensive Care and Trauma Management (Sektion NIS) of the German Trauma Society (DGU), Berlin, Germany

**Keywords:** Pelvic binder, Pelvic ring injury, Registries, Multiple trauma, Emergency medical services, Emergency treatment, Advanced trauma life support care

## Abstract

**Background:**

Pelvic Circumferential Compression Devices (PCCD) are standard in hemorrhage-control of unstable pelvic ring fractures (UPF). Controversial data on their usefulness exists. Aim of the study was to investigate whether prehospital application of PCCD can reduce mortality and transfusion requirements in UPF.

**Methods:**

Retrospective cohort study. From 2016 until 2021, 63,371 adult severely injured patients were included into TraumaRegister DGU^®^ of the German Trauma Society (TR-DGU). We analyzed PCCD use over time and compared patients with multiple trauma patients and UPF, who received prehospital PCCD to those who did not (noPCCD). Groups were adjusted for risk of prehospital PCCD application by propensity score matching. Primary endpoints were hospital mortality, standardized mortality rate (SMR) and transfusion requirements.

**Results:**

Overall UPF incidence was 9% (N = 5880) and PCCD use increased over time (7.5% to 20.4%). Of all cases with UPF, 40.2% received PCCD and of all cases with PCCD application, 61% had no pelvic injury at all. PCCD patients were more severely injured and had higher rates of shock or transfusion. 24-h.-mortality and hospital mortality were higher with PCCD (10.9% vs. 9.3%; *p* = 0.033; 17.9% vs. 16.1%, *p* = 0.070). Hospital mortality with PCCD was 1% lower than predicted. SMR was in favor of PCCD but failed statistical significance (0.95 vs. 1.04, *p* = 0.101). 1,860 propensity score matched pairs were analyzed: NoPCCD-patients received more often catecholamines (19.6% vs. 18.5%, *p* = 0.043) but required less surgical pelvic stabilization in the emergency room (28.6% vs. 36.8%, *p* < 0.001). There was no difference in mortality or transfusion requirements.

**Conclusion:**

We observed PCCD overuse in general and underuse in UPF. Prehospital PCCD appears to be more a marker of injury severity and less triggered by presence of UPF. We found no salutary effect on survival or transfusion requirements. Inappropriate indication and technical flaw may have biased our results. TR-DGU does not contain data on these aspects. Further studies are necessary. Modular add-on questioners to the registry could offer one possible solution to overcome this limitation. We are concerned that PCCD use may be unfairly discredited by misinterpretation of the available evidence and strongly vote for a prospective trial.

## Introduction

Pelvic ring injuries are potentially associated with life-threatening hemorrhage [1]. Exsanguination is the number one cause of preventable death after trauma and pelvic bleeding is among the top sources after blunt trauma [2].

Closed reduction and external fixation are standard procedures to control hemorrhage in complex pelvic ring injuries [3]. External fixators and C-Clamps are effective measures for bleeding control. Non-invasive external stabilization such as pelvic sheeting or use of pelvic binder were deemed similarly successful [4]. The mode of action of pelvic circumferential compression devices (PCCD) and their equivalence to invasive surgical techniques has been widely demonstrated [4–6]. There is a notion that by reducing the size of the bleeding spaces in the pelvis and retroperitoneum, hemostasis is achieved by self-tamponade [3]. Tan et al. 2010 conducted a prospective cohort study of 15 patients with pelvic fractures. They monitored physiological and radiological effects of PCCDs and showed significant improvement in hemodynamic function [7]. In another case series, Nunn et al. demonstrated marked improvement of hemodynamics with PCCD [8].

Emergency Medical Service (EMS) personnel is capable to apply non-invasive external fixation already in the field without any surgical capacities or skill. Over the last years, commercial PCCD became more and more popular as one non-invasive method to control hemorrhage in pelvic ring injuries. Available products are extremely user-friendly and are much easier to handle than improvised devices.

With the increasing popularity of PCCD and widespread use of non-invasive external stabilization, the question arises as to the actual benefit and effectiveness of this intervention. Several studies have looked for salutary effects of pelvic binders but most studies have failed to show life- and blood-saving effect [9–12]. However, experienced practitioners can usually recall anecdotal cases in which abrupt bleeding control was achieved by reduction and external stabilization of the pelvic ring.

We hypothesized that early application of PCCD in multiple trauma patients with unstable pelvic ring injury during the prehospital phase can prevent severe hemorrhagic shock and thus leads to a decrease in mortality and in transfusion requirements.

The aim of the study was to determine if prehospital PCCD application in patients with unstable pelvic injuries leads to improved survival and reduced transfusion requirements. For this, we compared patients with unstable pelvic fracture (UPF) who received PCCD during prehospital care with those who did not.

## Material and methods

This is a retrospective cohort study of data from the TraumaRegister DGU^®^ (TR-DGU) of the German Trauma Society (Deutsche Gesellschaft für Unfallchirurgie, DGU). The registry was founded in 1993. The aim of this multi-center database is a pseudonymous and standardized documentation of severely injured patients.

Data are collected prospectively in four consecutive time phases from the site of the accident until discharge from hospital: (A) Pre-hospital phase, (B) Emergency room and initial surgery, (C) Intensive care unit and (D) Discharge. The documentation includes detailed information on demographics, injury pattern, comorbidities, pre- and in-hospital management, course on intensive care unit, relevant laboratory findings including data on transfusion and outcome of each individual. The inclusion criterion is admission to hospital via emergency room with subsequent ICU/IMCU care or reach the hospital with vital signs and die before admission to ICU.

The infrastructure for documentation, data management, and data analysis is provided by AUC—Academy for Trauma Surgery (AUC—Akademie der Unfallchirurgie GmbH), a company affiliated to the German Trauma Society. The Committee on Emergency Medicine, Intensive Care and Trauma Management (Sektion NIS) of the German Trauma Society provides the scientific leadership. The participating hospitals submit their data pseudonymous into a central database via a web-based application. Scientific data analysis is approved according to a peer review procedure laid down in the publication guideline of TraumaRegister DGU^®^.

The participating hospitals are primarily located in Germany (90%), but a rising number of hospitals of other countries contribute data as well (at the moment from Austria, Belgium, China, Finland, Luxembourg, Slovenia, Switzerland, The Netherlands, and the United Arab Emirates). Currently, more than 28,000 cases from almost 700 hospitals are entered into the database per year.

Participation in TraumaRegister DGU^®^ is voluntary. For hospitals associated with TraumaNetzwerk DGU^®^, however, the entry of at least a basic data set is obligatory for reasons of quality assurance (www.traumaregister-dgu.de).

The present study is in line with the publication guidelines of the TraumaRegister DGU^®^ and registered as TR-DGU project ID 2020–028.

### Selection of cases

This study aimed to analyze effects of prehospital PCCD-application in patients with multiple trauma and proven UPF.

We included adult patients from TR-DGU admitted primarily to a German hospital between 2016 and 2021. All inter-hospital transfers were excluded.

To describe PCCD use in patients with multiple trauma, we included all cases with complete data on PCCD use. PCCD use as an item was introduced to TR-DGU with the 2015 update [13].

UPF was defined according to the Abbreviated Injury Scale (AIS): partially unstable pelvic ring fracture like open book fractures, symphysis pubis separation, or lateral compression, with or without relevant blood loss (AIS codes 85616x.x), and unstable pelvic ring fracture, like vertical shear fractures, or dislocation, with or without relevant blood loss (AIS codes 85617x.x). TR-DGU relies on a short version of the AIS (Version 2005 Update 2008) to classify injury severity. This version does not provide sub-classification of pelvic fractures according to Tile. The AIS of these fractures range from 3 to 5. Acetabular fractures were excluded.

**Fig. 1:**
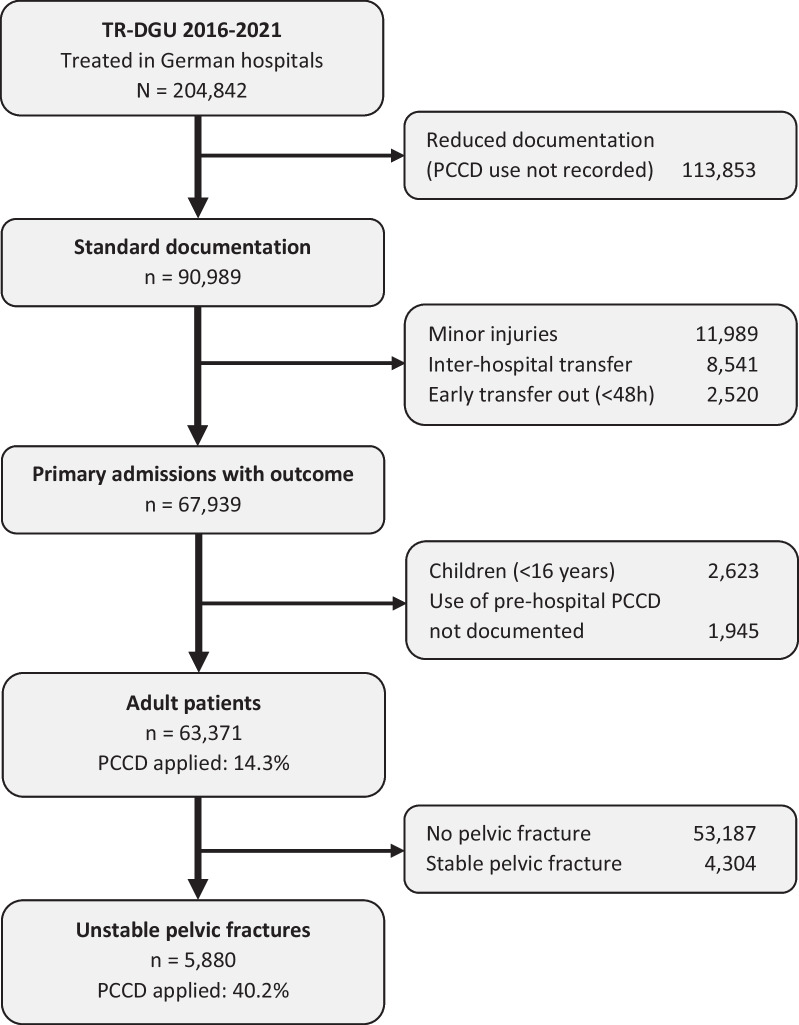
Case selection flow chart

For details of patient selection see the flow sheet in Fig. 1.

### Primary endpoint

Hospital mortality was defined as any death during acute hospital stay. Early mortality was defined as death within the first 24 h after hospital admission. Predicted mortality was calculated by the Revised Injury Severity Classification score, version 2 (RISC II) [14]. Standardized Mortality Rate (SMR) is the quotient of observed and predicted mortality rate. A SMR below 1.0 indicates a favorable outcome since mortality was lower than expected. Mortality rates and SMR were reported with 95% confidence intervals (CI95).

### Definition of other variables

We defined prehospital shock as the first systolic blood pressure taken on scene as of 90 mmHg or less (sysBP ≤ 90 mmHg) and prehospital loss of consciousness as the first GCS score taken on scene as of 3 to 8 points on the Glasgow Coma Scale (GCS).

Road traffic accident included all traffic related accidents such as automobile, motorcyclists, bicycle or hit pedestrians.

Mass transfusion was defined as administration of 10 or more units of packed red blood cells (pRBC).

### Propensity score matching

Protocols and procedures for the prehospital use of PCCD are not standardized and it is unclear when and why a PCCD was applied to the patient or not. Since it is very likely that there are different perceptions among EMS providers as to whom a PCCD should be applied, a matched pairs analysis was performed based on a propensity score. The propensity score is equivalent to the probability that a PCCD will be applied. We used age, sex, prehospital crystalloid volume, catecholamine administration, intubation, chest tube application, first prehospital blood pressure and first prehospital GCS, use of tranexamic acid, injury mechanism, destination (Level 1 trauma center) and type of transport (ground; helicopter) in a multiple logistic regression analysis to predict prehospital PCCD use. Non-significant predictors were excluded from the model and categories with similar effects were merged. To each case with PCCD (PCCD) we matched one case with an identical (rounded percentage) propensity score but without application of PCCD (noPCCD).

### Statistical analysis

Data of eligible cases were extracted from the TR-DGU database and analyzed with SPSS (Statistical Package for the Social Sciences, Version 28, IBM Inc., Armonk, NY, USA).

We performed three analysis: 1) Use of PCCD over time 2) PCCD use in patients with UPF and 3) Comparison of propensity score matched pairs of patients with UPF and with or without PCCD application.

Unless otherwise specified, all numbers are given as mean with standard deviation (SD) or as a percentage along with the number of cases. In case of obviously skewed distributions, median with interquartile rage (IQR) will be given instead.

Where appropriate, we performed the Mann–Whitney U-test for metric data and Chi-squared test for categorical data. A *p *value < 0.05 was considered statistically significant. However, due to the large sample size significant differences should always be checked for relevance. The dimension of the difference should therefore be the primary guide for interpretation.

## Results

### Use of PCCD over time

**Fig. 2:**
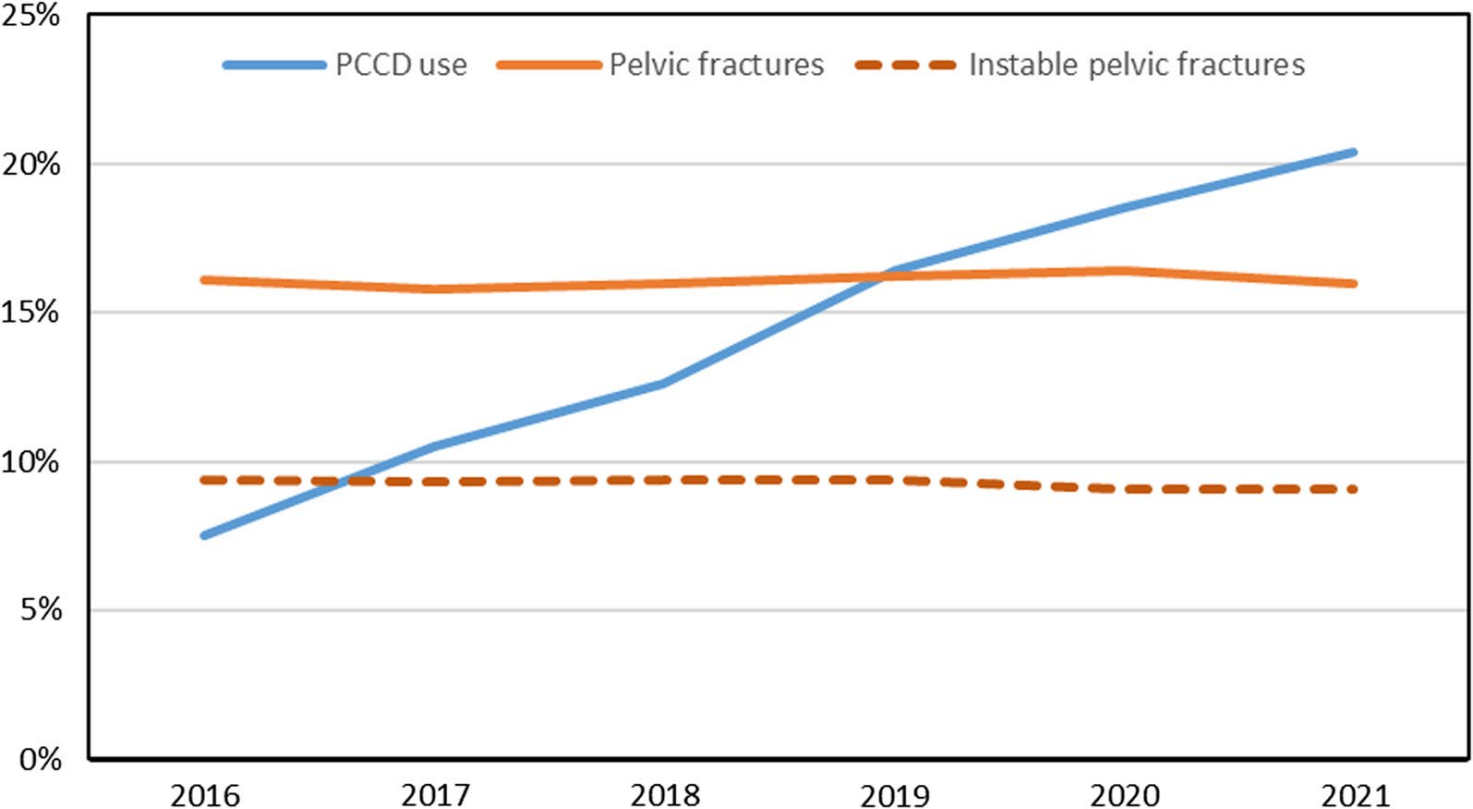
Prevalence of pelvic fractures, unstable pelvic fractures, and prehospital PCCD use in adult trauma patients (TR-DGU 2016–2021, n = 63,371) over time

Between 2016 and 2021, there were 63,371 cases with information on whether patients received a PCCD or not. This information was missing in 3% of all cases. Of these, 9,085 were treated with prehospital PCCD (14.3%). There was a marked increase in prehospital PCCD use over time. While in 2016 the rate of prehospital PCCD use was 7.5%, there is a documented rate of 20.4% in 2021. The proportion of pelvic fractures (about 16%) as well as UPF (about 9%) remained stable over time (Fig. 2). However, the majority of PCCDs was applied to patients without an unstable pelvic fracture (n = 6719, 74%), or even without any pelvic fracture (n = 5530, 61%).

### PCCD use in patients with unstable pelvic injury

Among patients with UPF (n = 5880), there were 2366 cases, who received a PCCD during prehospital care (40.2%). The use of prehospital PCCD increased from 26% in 2016 to 52% in 2021.

Characteristics of patients with and without use of PCCD are given in Table 1.

**Table 1:** Characteristics of patients with unstable pelvic fractures, with and without prehospital PCCD application

	noPCCD N = 3514	PCCD N = 2366	Total N = 5880	*p *value
Male sex [%]	61.3	66.0	63.2	< 0.001
Age [years] ± SD	53 ± 21	48 ± 19	51 ± 20	< 0.001
Penetrating mechanism [%]	1.2	1.5	1.3	0.27
**Type of accident**				
Road traffic accident [%]	54.2	58.2	55.8	0.002
High fall > 3 m [%]	29.0	32.2	30.3	0.007
Low fall < 3 m [%]	12.1	3.8	8.8	< 0.001
ISS [points] ± SD	29.3 ± 14.6	32.2 ± 16.1	30.4 ± 15.3	< 0.001
Shock on scene [%]	15.5	24.6	19.1	< 0.001
Unconscious on scene [%]	17.0	21.0	18.6	< 0.001
Proximal femur fracture [%]	17.2	22.5	19.3	< 0.001
**Prehospital interventions**				
Intubation [%]	27.0	39.6	32.0	< 0.001
Catecholamine administration [%]	12.9	24.9	17.7	< 0.001
Cardio-pulmonary resuscitation [%]	4.2	7.7	5.6	< 0.001
Tranexamic acid administration [%]	11.8	35.0	21.1	< 0.001
Crystalloid volume given [ml], median (IQR)	500 (500–1000)	1000 (500–1500)	500 (500–1000)	< 0.001
More than 1000 ml of crystalloids given [%]	16.2	29.8	21.6	< 0.001
On scene time [minutes] ± SD	30 ± 16	33 ± 17	31 ± 17	< 0.001
Transportation by helicopter [%]	26.6	40.5	32.3	< 0.001
**Hospital care**				
Treatment in Level 1 hospital [%]	81.6	90.4	85.2	< 0.001
Blood transfusion [%]	23.7	35.5	28.4	< 0.001
Mass transfusion [%]	4.7	8.0	6.0	< 0.001
Surgical stabilization of pelvis before ICU admission [%]	24.8	38.2	30.3	< 0.001
Angio-embolization [N]	1.4	1.8	1.5	0.270
Length of stay on ICU [days], median (IQR)	4 (1–12)	5 (2–17)	4 (2–14)	< 0.001
Length of stay in hospital [days], median (IQR)	18 (10–29)	20 (11–34)	19 (10–31)	< 0.001

IQR = Interquartile range

Patients with prehospital PCCD were on average five years younger. They were more severely injured, with higher ISS and higher rates of shock and unconsciousness in the field. Other prehospital interventions were also carried out more often on PCCD cases than noPCCD cases. Of all patients, 95.2% were brought to a Level 1 trauma center. Patients with prehospital PCCD application were referred to Level 1 trauma centers more often than noPCCD. On-scene time was on average 3 min longer for PCCD patients (not adjusted for severity). They also required more blood transfusions and more often surgical stabilization of the pelvis before admission to ICU (Table 1). Of those with noPCCD, 19.5% received a PCCD upon admission to the ER.

Early mortality was higher with PCCD (10.9% vs. 9.3%; *p* = 0.033). The observed hospital mortality rates were slightly higher but not significantly different in patients with PCCD as compared to noPCCD (17.9% vs. 16.1%, *p* = 0.070). Predicted mortality by use of RISC II Score with PCCD was 18.9% and with noPCCD 15.4%. Thus, hospital mortality in the PCCD group was 1% lower than predicted while it was higher than expected in the noPCCD group (+ 0.7%). The SMR was in favor of PCCD application in patients with UPF but also failed to reach statistical significance (0.95 vs. 1.04, *p* = 0.101). For details, see Table 2.

**Table 2:** Hospital mortality and risk of death in patients with and without prehospital PCCD

	noPCCD N = 3514	PCCD N = 2366	*p *value
Mortality in the first 24 h [%]	9.3	10.9	0.036
Hospital mortality [%] (CI95)	16.1 (14.9–17.3)	17.9 (16.3–19.4)	0.070
Risk of death based on RISC II score [%] (CI95)	15.4	18.9	0.033
Standardized Mortality Ratio (CI95)	1.04 (0.97–1.12)	0.95 (0.86–1.03)	0.101

CI95 = 95% confidence interval

### Comparison of propensity score matched pairs of patients with UPF and with or without PCCD application

For 5726 patients (97.4%) a propensity score could be calculated ranging from 9–88%. The most important predictor for the application of a PCCD was the administration of tranexamic acid (OR 2.91, Table 3).

**Table 3:** Results of logistic regression analysis for the propensity score predicting pehospital PCCD use

Predictor	Prevalence	Odds ratio	95CI
Younger age (< 60 years)	3709	1.48	1.31–1.68
Systolic BP (reference: 110 + mmHg)			
0 mmHg / CPR	129	2.09	1.39–3.16
1–109 mmHg	1756	1.26	1.11–1.43
Unconsciousness on scene	1033	0.67	0.57–0.80
Prehospital volume (reference ≤ 500 ml)			
Up to 1000 ml	1595	1.4	1.22–1.59
Up to 2000 ml	1003	1.52	1.29–1.79
More than 2000 ml	250	1.75	1.30–2.36
Tranexamic acid administration	1212	2.91	2.51–3.37
Catecholamine administration	991	1.4	1.17–1.68
High energy trauma (traffic, high fall)	4925	1.42	1.20–1.70
Transportation by helicopter	1813	1.26	1.11–1.43
Destination level 1 hospital	4877	1.62	1.36–1.93

Regression is based on 5726 cases with complete data for all predictors. Nagelkerke’s R^2^ = 0.157

Based on the rounded propensity score, 1860 pairs could be matched (n = 3720). Patients in both groups were of similar age, had comparable mechanism of injury and injury severity. Both groups received similar prehospital treatment. Length of stay on ICU and in hospital was similar in both groups. For details, see Table 4.

**Table 4:** Characteristics of patients with unstable pelvic ring injury after propensity score matching

	noPCCD N = 1860	PCCD N = 1860	*p *value
Male sex [%]	63.0	65.6	0.10
Age [years] ± SD	49.3 ± 20.0	48.5 ± 19.5	0.31
Penetrating trauma mechanism [%]	1.5	1.2	0.47
High energy mechanism—traffic; high falls [%]	90.8	89.1	0.10
ISS [points] ± SD	31.1 ± 14.8	30.7 ± 15.7	0.13
Shock on scene [%]	21.1	20.0	0.47
Unconscious on scene [%]	20.4	18.7	0.21
proximal femur fractures [%]	19.5	19.8	0.836
**Prehospital interventions**			
Intubation [%]	35.2	32.2	0.066
Catecholamine administration [%]	19.6	18.5	0.043
Tranexamic acid administration [%]	20.9	21.7	0.55
Crystalloid Volume given [ml], median (IQR)	1000 (500–1000)	1000 (500–1000)	0.50
More than 1000 ml of crystalloids given [%]	23.4	23.7	0.85
On scene time [minutes] ± SD	33 ± 17	31 ± 17	0.74
Transportation by helicopter [%]	35.2	34.9	0.84
**Hospital treatment and outcome**			
Treatment in Level 1 hospital [%]	88.7	88.8	0.92
Blood transfusion [%]	29.1	31.2	0.17
Number of pRBC, if transfused [N]	7.1	6.9	0.15
Mass transfusion [%]	6.0	6.6	0.48
Surgical stabilization of pelvis before ICU admission [%]	28.6	36.8	< 0.001
Angio-embolization [%]	1.7	1.6	0.896
Length of stay on ICU [days], median (IQR)	5 (2–15)	5 (2—15)	0.73
Length of stay in hospital [days], median (IQR)	20 (11–31)	19 (11—32)	0.15

There was no relevant difference in blood transfusion rates. Patients with PCCD needed more often surgical stabilization of the pelvis before ICU admission than noPCCD (36.8% vs. 28.6%, *p* < 0.001). Of those with noPCCD, 21.1% received a PCCD in the ER upon admission (Table 4). Hospital mortality and early mortality was marginally higher in the noPCCD group but these differences were not statistically different. Risk of death estimation based on RISC II also suggested an increased risk in the noPCCD group compared to PCCD but that was not statistically significant (0.5% higher, *p* = 0.20). SMR indicated no advantage associated with PCCD-use (Table 5).

**Table 5:** Hospital mortality and risk of death in patients with and without prehospital PCCD after propensity score matching

	noPCCD N = 1860	PCCD N = 1860	*p *value
Mortality in the first 24 h [%]	9.9	8.9	0.26
Hospital mortality [%] (CI95)	16.8 (15.1–18.5)	15.5 (13.9–17.2)	0.29
Risk of death based on RISC II score (%)	17.1	16.6	0.20
Standardized mortality ratio (CI95)	0.98 (0.89–1.08)	0.94 (0.84–1.04)	0.50

CI95 = 95% confidence interval

## Discussion

The use of PCCD has increased significantly over time, while the rate of UPF remained constant. At the same time, the majority of PCCD applications (61%) were on patients who did not even suffer a pelvic fracture at all. Likewise, many patients with UPF did not receive PCCD (59.8%).

Similar findings were reported elsewhere. For example, in a prospective multi-center trial from Germany found that 65.4% of all patients suspicious of pelvic ring injury received external pelvic stabilization at some point during the prehospital or early hospital phase. Of these, only 37.3% had a UPF. However, 34.7% of these patients had UPF but no form of external pelvic stabilization at all [15]. Another study found a similar large proportion of patients with pelvic ring injuries who had no binder applied (44.8%) and of whom 20% had an unstable injury [16].

The true reasons for PCCD omission despite UPF remain elusive. It is likely, that local EMS protocols may vary considerably on when to apply PCCD. It is possible that the rescuers applied PCCD as a precaution in view of the assumed severity of the injury, just to make sure that a possible UPF was adequately addressed even without a concrete clinical findings. It also may be that in some EMS, PCCD are not available on a regular basis. In the unadjusted study population, observed hospital mortality with PCCD was 1.8% higher and transfusion rates and rates of massive transfusion were increased compared with noPCCD. The expected mortality of the PCCD group was 3.5% higher than in the noPCCD group. Patients with PCCD also received more prehospital interventions such as fluid resuscitation, intubation, catecholamines and administration of tranexamic acid (Table 1). There was also a higher proportion of low falls and mean age was higher. This finding may indicate a larger proportion of elder patients in this group who sustained low energy trauma. Age is a risk factor for increased mortality after trauma [14]. These findings indicate that there was a higher degree of injury severity and substantial hemorrhagic shock.

With PCCD, the observed mortality was 1% lower than expected by RISC II in the PCCD-group indicating salutary effects, while observed mortality with noPCCD was 0.7% higher than expected which might indicate adverse outcome. Both observations may suggest life-saving effect of PCCD. However, the SMR was only slightly in favor of PCCD application and did not reach the level of statistical significance (Table 2).

When we controlled for group differences by propensity score matching, PCCD use did neither affect rates of transfusion, massive transfusion or number of units of pRBC. Rate of catecholamine use in noPCCD was higher (Table 4), which might indicate more severe hemorrhagic shock. However, there was no statistically significant advantage of PCCD-use on survival (Table 5).

Thus a 6 years data collection in an 80 million population (Germany) revealed not enough evidence to prove that prehospital PCCD application reduced mortality or prevented severe hemorrhagic shock in multiple trauma patients with UPF.

Bangura et al. compared PCCD application in the prehospital phase vs. PCCD application in the ED and also could not find any benefit between early and late intervention [17]. Other authors have reported similar results [9–12]. In many of these studies the definition of the indication, type of conduct and timing of the intervention were ambiguous.

Two studies by Hsu et al. and Rungsinaporn et al. demonstrated positive therapeutic effects in patients with PCCD. Hsu et al. found shorter duration of hospital and ICU stay, improved survival and lower mean blood transfusion volume when patients with suspected pelvic injury were treated with PCCD early after ER admission. Historic cases in whom PCCD was applied after clinical or radiological confirmation of a pelvic fracture served as control [18]. Rungsinaporn et al. who also assessed blood saving effects from early PCCD application in the ER, showed that PCCD on patients suspicious of pelvic injury lead to considerably lower number of pRBC transfusions in the early PCCD group compared to historic controls where PCCD was only applied after radiological confirmation of the pelvic fracture [19]. This advantage could be due to a time difference of approx. 24 min, which the PCCD was applied earlier in the intervention group (*p* = 0.001). Both studies advocate for early PCCD use based on clinical suspicion alone, which underlines the meaningfulness of the measure. They also put major emphasize on clinical examination and proper indication, too. Nevertheless, they are in contrast with other work—including ours.

In view of these results, the question arises as to why PCCD do not bring the promised benefits. PCCD are supposed to provide mechanical stabilization of the unstable pelvic ring injury. Its purpose is to control bleeding. We hypothesized that PCCD application in UPF can reduce blood loss and thus reduces transfusion requirements and improves survival. Consequently, recognition of UPF with pelvic haemorrhage are prerequisite for identifying patients who could benefit from the application.

Recognition of UPF based on physical examination alone (without imaging) is a challenge. Studies indicate that injuries to the pelvic ring often go unrecognized in the prehospital phase [15, 20, 21]. Physical findings (such as spontaneous pain, pain to pressure on careful palpation and visible external injuries such as hematoma, perineal ecchymosis, wounds, open fractures, visible deformity, leg length discrepancy, malrotation of the leg, diastasis of the symphysis, and bleeding from the urethra, vagina, or anus) were indicative for pelvic ring injury [15] and may guide providers to the correct diagnosis. However, assessment of findings may be complicated by impaired consciousness or when patients are unable to differentiate their pain. In our study, 19.1% had a prehospital GCS of 8 points or less and 32% were intubated (Table 1).

Pehle et al. found that independent from fracture classification, only those fractures that actually feel unstable during mechanical stability testing have a serious risk of bleeding with increased lethality and higher rates of blood transfusion or emergency surgery [22]. Positive pelvic compression test was an inclusion criterion in the study by Rungsinaporn et al. who showed that PCCD use reduced transfusion requirements [19]. They may have succeeded in enrolling only patients with severer bleeding from UPF in whom the intervention could exert positive effects. TR-DGU does not provide data on mechanical stability testing and thus, we cannot use it for inclusion or exclusion criteria. Radiologic imagining is unavailable during the prehospital phase and radiologic fracture classification alone is a poor predictor for bleeding [6, 22].

According to the literature, not all pelvic ring injuries are associated with severe bleeding [6, 22]. Pelvic fractures that are complicated by associated injuries to vessels, nerves, soft tissues, and internal organs of the small pelvis are classified as complex pelvic injuries [23]. These are potentially life-threatening pelvic injuries with high mortality rate of up to 20% [24]. Gänsslen et al. reported life-threatening hemorrhage in about 1–2% of all pelvic fractures and not all hemorrhages are treatable with the concept of external mechanical stabilization of the pelvic ring [3]. Therefore, benefits of PCCD anecdotally experienced in individual cases or selected case series [7, 8] can probably only be demonstrated in highly selected subgroups. Valid estimation of blood loss in patients with multiple trauma, who may have multiple, co-existing sources of bleeding, is difficult and thus is prone to error. It is possible that misclassification has occurred and that patients who did not actually bleed from their pelvic injury received PCCD and thus were included into the PCCD group of our study. One cannot expect positive effects from PCCD-application in patients that do not bleed from the pelvis or whose source of bleeding cannot be controlled by PCCD. This may have biased our results and has to be considered as a universal confounder to all retrospective studies. Therapeutic effect of PCCD might be more obvious in patients with hemorrhagic shock or mechanical instability. This hypothesis deserves further attention in another analysis.

In many studies, that failed to show positive effects from PCCD, the time point of intervention was not standardized [9, 10, 12, 17]. In some studies, PCCD was placed during initial assessment and management in the emergency room (ER) [11, 18, 19] presumably too late to prevent blood loss and subsequent severe hemorrhagic shock after significant blood loss already occurred during the prehospital phase.

We discussed other possible reasons for treatment effect bias. Three of these seem to us to be of particular interest: First, incorrect positioning of PCCD. Second, cause of the hemorrhagic shock is not of pelvic origin or bleeding is inaccessible to PCCD. Third, PCCD aggravated bleeding because specific fracture types are possible contraindication.

Maybe, PCCD-deployment was technically incorrect in many cases and therefore could not develop the full effect. Unsatisfactory positioning of the pelvic binder is a common problem. One study reported optimal placement of pelvic binders in just 56.6% while 43.5% were positioned suboptimal with 39.7% placed too high and 3.8% placed too low [25]. Another study found 49.1% in a satisfactory position; 40.9% were high and 10% were in a low position [16]. Even trained teams do not sufficiently master PCCD deployment! In a prospective study on the prehospital use of pelvic binders with trained users, only 80% of the binders were placed correctly [26]. Given the application of pelvic binders is aimed at improving hemodynamic management of unstable pelvic injuries it is important to ensure correct fit for optimal efficacy and reduction of complications [25].

We have examined patients with multiple trauma. One possible explanation is that more than one source of bleeding may contribute to blood loss and severity of shock. Occult blood loss in compartments other than pelvis such as chest, abdomen and extremities (e.g. femur fractures) may complicate shock but may be inaccessible to bleeding control with PCCD. Perhaps the increase in mean arterial pressure after PCCD increased bleeding in other regions.

Tile considered only open book type fractures suitable to be treated with a strap or pelvic sling because he feared that they would tend to increase the deformity in the treatment of the lateral compression (LC) injuries [27]. Consequently, the Western Trauma Association advocated that circumferential pelvic sheeting and binders are contraindicated in LC fractures [28]. However, in both studies reporting favorable effects from PCCD, LC-type fractures were the most common at 70–80% [18, 19], making this concern unwarranted.

### Limitations

This study has several methodological limitations.

The TR-DGU data set does not included information on mechanical stability or leading source of major bleeding. Since UPF alone is no indicator of pelvic hemorrhage. It is possible that we have therefore included many patients for whom the PCCD could not be of any use.

The survival of a complex pelvic injury with massive bleeding is largely determined by the measures taken to control the bleeding. Unfortunately, TR-DGU contains only rough information on those measures. For example, we have no information about the frequency of pelvic packings for bleeding control.

TR-DGU does not comprise patients who bleed to death in the field. Only those who arrived to the hospital are included. We may have missed cases who would have benefited from early PCCD application but bled to death on scene without it. We cannot answer the question if prehospital death was influenced by PCCD-application.

TR-DGU is not only missing important information on indication but also on technical implementation of the intervention. TR-DGU does neither specify the type or technique of PCCD nor if it was applied orderly. In some cases, even improvised pelvic binder or pelvic sheeting may have been applied. Different devices or technique may have different effectiveness. This limitation is certainly true for all registry-based analysis of emergency interventions and we have to admit that our study has reached the limit of statistical robustness of our registry data.

In order to study effectiveness of emergency interventions, registries can only be used for hypothesis generation. There is a lack of details regarding the identification and technical implementation for assessing whether the measure has any prospect of success. To eliminate this limitation from future studies, we propose to extend the standard data set of the registry with modular add-on questionnaires on specific interventions of interest, which can be used to collect data for a limited period of time. This limits on the one hand the disproportionate documentation effort and on the other hand allows an increase in scientific knowledge. Only prospective studies with precise protocols for indication and management will be able to answer the question if prehospital PCCD have salutary effects in complex pelvic fractures for good.

## Conclusion

We observed high rates of overuse of PCCD in severely injured patients without any pelvic injury and high rates of underuse in patients with proven UPF during prehospital care.

Prehospital PCCD application on patients with multiple trauma and proven unstable pelvic fracture show no statistical benefit for improved survival or blood saving effects.

Our pathophysiological concepts on complex pelvic trauma may be wrong or the results of our study may be biased because registry data does not provide information on indication and technical implementation of the intervention. Findings may also be biased because a veritable number of cases with UPF have no significant bleeding. With biased data we won´t be able to find out if the intervention does any good or harm. This is subject to all retrospective studies on PCCD effectiveness.

Our findings are in some ways dangerous, as clinical experience shows that in some cases external non-invasive stabilization is a simple and important measure to save lives. In the light of our results and similar reports, we are concerned that the procedure may be unfairly discredited by misinterpretation of the available evidence and from flawed understanding of evidence based medicine.

Further studies are needed to get to the heart of this therapeutic procedure. We strongly vote for a prospective trial with clearly defined indication, method and protocol in order to assess PCCD effectiveness in multiple trauma patients. Modular add-on questioners to the registry could offer one possible approach.

## Data Availability

The datasets generated and/or analyzed during the current study are not publicly available due to the regulations of TraumaRegister DGU^®^ (see Publication guideline TraumaRegister DGU^®^, www.traumaregister-dgu.de). Requests for data analysis from third parties (e.g. research institutes, industry) require the basic approval of the steering group of the TraumaRegister DGU^®^ (see Review Board /Review Process). All data generated or analyzed during this study are included in this published article.

## Abbreviations

EMS: Emergency medical service (EMS)
PCCD: Pelvic circumferential compression devices
pRBC: Packed Red Blood Cells
TR-DGU: TraumaRegister DGU^®^ of the German Trauma Society
CI95: 95% Confidence interval
IQR: Interquartile range
